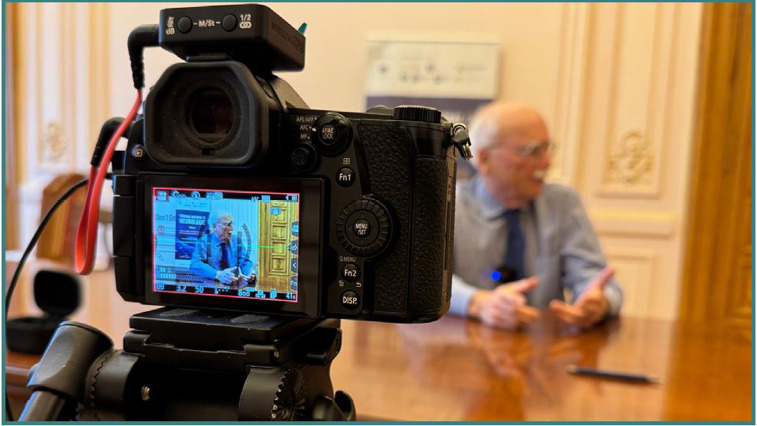# Interview with Professor Marc Fisher, past president of the World Stroke Organisation, at the 3^rd^ National Neurology Forum in Bucharest, Romania

**DOI:** 10.25122/jml-2025-1002

**Published:** 2025-05

**Authors:** Stefana-Andrada Dobran, Alexandra Gherman

**Affiliations:** 1 RoNeuro Institute for Neurological Research and Diagnostic, Cluj-Napoca, Romania; 2 Sociology Department, Babes-Bolyai University, Cluj-Napoca, Romania

**Interviewee: Professor Marc Fisher**

**Interviewer: Stefana-Andrada Dobran**

Professor Marc Fisher, a leading expert in neurology and past president of the World Stroke Organisation (WSO), joined the 2025 National Neurology Forum in Romania to share his insights on the event’s role in driving systemic change and improving patient outcomes. The third edition, held on April 10-11 at the Patriarchate Palace in Bucharest, brought together neurologists, policymakers, patient advocates, and industry leaders to translate strategic visions into actionable reforms for cardiovascular and cerebrovascular health. With workshops addressing stroke, neurodegenerative diseases, epilepsy, and rare neurological disorders, the scientific event focuses on reshaping Romania’s healthcare landscape.

The National Neurology Forum is organized with the help of the Foundation of the Society for the Study of Neuroprotection and Neuroplasticity, the Romanian Ministry of Health, the Romanian Neurology Society, Carol Davila University of Medicine and Pharmacy of Bucharest, Iuliu Hațieganu University of Medicine and Pharmacy of Cluj-Napoca, and George Emil Palade University of Medicine, Pharmacy, Science, and Technology of Târgu Mureș.

The Forum is endorsed by the European Federation of Neurorehabilitation Societies (EFNR) and will have the Academy for Multidisciplinary Neurotraumatology (AMN) as a future development partner.

**S.D.: Dear Professor Fisher, what are your overall impressions of the National Neurology Forum, an event dedicated to turning strategies into tangible solutions for brain health?**

M.F.: The concept of the meeting is to discuss various areas in neurology and how to improve the care of patients with very particular disorders as well as develop a national plan, especially for stroke, to improve patient care, education, and access. I think this approach will have a great impact, at least in the stroke field, in improving the ability of patients to get acute care, prevention, and rehabilitation. I think the concept is really good.

**S.D.: As the past president of the World Stroke Organization (WSO), what is your advice concerning global stroke care strategies that Romania could adopt to strengthen the national strategy for combating cardiovascular and cerebrovascular diseases?**

M.F.: That's a broad question. Seeing today’s presentations, it's good that there has been a substantial increase in the percentage of patients who are getting acute stroke therapy, both with thrombolysis and thrombectomy. However, there is still a long way to go. There are several components that need to be addressed to increase patient awareness. *Patient education* is an important aspect of improving stroke care so that patients recognize symptoms of stroke early and do not delay seeking care. They need to get evaluated and treated as quickly as possible. Acute stroke therapy is time-sensitive; for many patients, the faster they get treated, the better the chances of a good outcome. Another important aspect of patient education is increasing awareness of stroke risk factors such as high blood pressure, high cholesterol, smoking, and diabetes, because by impacting the risk factors you reduce risk. The risk factors for ischemic and hemorrhagic stroke are very similar to those for cardiovascular disease. There is significant overlap - for example, aggressively treating high blood pressure (the target that we now use for patients with a stroke or a heart attack is less than 130 over 80 in most cases), lowers the risk for both conditions. There are many commonalities between neurologists who treat stroke and cardiologists who treat ischemic heart disease.

Those are the two big areas that we focus on at WSO because rehabilitation, which you will hear more about later, while essential, comes later in the process. So, the two main pillars are (1) *prevention and patient education leading to the reduction of stroke risk*, and (2) *the availability of acute treatment and ensuring timely access for patients to the medical system*.


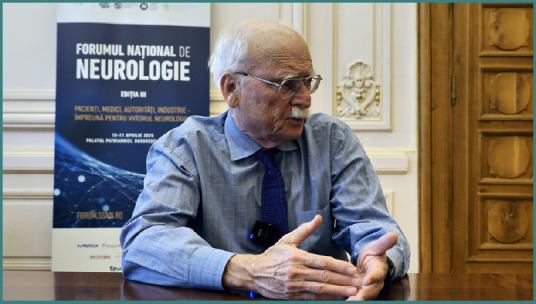


**S.D.: The workshops here aim for tangible deliverables. Could you share a successful example of cross-sector collaboration in stroke prevention?**

M.F.: As I mentioned earlier, there is significant overlap between cardiology and stroke neurology. In stroke care, we are dependent on having a close collaborative working relationship with cardiologists. What we have been doing at my hospital in Boston for quite a while now is that we hold a monthly case conference with interventional cardiologists to discuss patients who are being considered for closure of *patent foramen ovale* which was the cause of a stroke, or people who need another procedure called *left atrial appendage closure* because they have atrial fibrillation and are not good candidates for anticoagulation. We have been doing that for a long time and we are more aligned now about which patients should have a certain procedure, and what tests are needed. Then, for my patients, once they are discussed, I will contact them and let them know the recommendations decided at the meeting.

Another area where we work very closely with cardiologists is in the case of a substantial percentage of ischemic stroke patients where, despite reasonable testing, we still don't know what the underlying cause was. We have a term for that – *embolic stroke of undetermined source*. Cardiac testing is crucial when we don't actually know the cause; we do echocardiograms, both transthoracic and transesophageal, which the cardiologists interpret, followed by cardiac monitoring. So, they are involved with the reading and interpretation of the cardiac monitors. Therefore, we truly need their help in trying to find cardiac sources for embolization of the brain. That is a good example of how these two disciplines work together and I hope that the effort here is going to have a similar effect, that there will be close relationships between the two disciplines.

**S.D.: From your experience, what are the most effective strategies to reduce symptom-to-door time?**

M.F.: That is a tough question. There are two big strategies that come to mind. First – as we’ve already discussed, *patient education* to ensure symptoms aren’t ignored. That needs to happen on a national level, with media in this country advertising information, hopefully for free, about stroke symptoms and what patients should do if they think they are having one (e.g. don't go back to bed when the arm is numb and weak thinking the symptoms are going to go away). The patients need to know to access the medical system. And there are various ways that that can be done. It’s important to not delay seeking care. Typically, in the U.S. we advise calling an ambulance right away to take them to the nearest hospital. Patient education about stroke symptoms is critical to ensure people don't ignore the signs and get help quickly.

The second strategy is *organizing healthcare delivery services*. This is a major focus of this conference – creating a national strategy for patients with acute ischemic stroke or even intracerebral hemorrhage (where there is no direct treatment yet) so when they access the system, the system is mobilized to take care of them as quickly and efficiently as possible. I will give an example of something that you could try to implement, because based on what we have discussed, it could be valuable here: telemedicine. Telemedicine is an important technology that has been used in stroke for quite a while. My hospital, for example, is connected to a network of smaller regional hospitals. When a suspected stroke patient arrives at one of their emergency rooms (ERs), they will notify the on-call trainee doctor that works with me. After discussing the case with the ER doctor, if we decide that a telemedicine consult is appropriate, then we are then able to do that through our computers. We can talk to and examine the patient with the help of the staff in the ER, as well as look at the imaging that has been done on this patient. It allows the relatively small number of stroke specialists to offer their expertise to smaller hospitals, many of which do not have a neurologist, let alone a stroke specialist. We can quickly decide whether the patient is an appropriate candidate for a clot-dissolving treatment, thrombolysis, or needs to be transferred to a bigger center for a clot removal procedure called thrombectomy.

Currently, I don't think this approach is widely implemented in Romania, and I would strongly encourage this forum and its supporting organizations to consider organizing telemedicine services. This would help expand the patient pool that will access acute treatments. In this way, even small hospitals in remote parts of the country could consult with experts to determine the best course of action. Another critical aspect is the *transportation system* - how to efficiently transfer a patient from outlying hospitals to a big center if they need thrombectomies. This requires organized networks. Given Romania’s road infrastructure where transport can be slow, helicopter transport is going to be necessary in many cases.

While significant progress has been made here in the last years, there is still a long way to go. And I know the plan is to expand patient evaluation and acute treatment and the growing number of patients receiving these treatments is already leading to better outcomes.